# EncoderMap III: A Dimensionality Reduction Package for Feature Exploration in Molecular Simulations

**DOI:** 10.1021/acs.jcim.5c00887

**Published:** 2025-08-20

**Authors:** Kevin Sawade, Tobias Lemke, Christine Peter

**Affiliations:** Department of Chemistry, 26567 University of Konstanz, Universitätsstr. 10, D-78457 Konstanz, Germany

## Abstract

EncoderMap is a dimensionality reduction method that is tailored for the analysis of molecular simulation data. It relies on a neural network autoencoder architecture augmented with an additional multidimensional scaling (MDS)-like loss term. Due to this additional cost function between the high-dimensional input and the latent space, EncoderMap emerges as a method that has advantages over other dimensionality reduction methods and straightforward autoencoders alike. In particular, the low-dimensional projections created by EncoderMap are guided by the MDS-like cost function adopted from the sketch-map algorithm, which produces projections with a better correlation between high- and low-dimensional similarities and dissimilarities. EncoderMap has been successfully applied to a wide range of molecular dynamics data. Here, we present a new version of the EncoderMap package. Porting EncoderMap to version 2 of the TensorFlow library not only ensures that it can be used on modern computers but most importantly allowed for the introduction of new features and a wide range of customization options (e.g., user-defined custom loss functions). We introduce new visualization capabilities and encoding/decoding features and make EncoderMap modular so that researchers can better understand the training process. Most of these additions can be used on general high-dimensional data, while some specifically aid in working with biomolecular simulation data. Furthermore, we added the possibility to provide sparse inputs to EncoderMap’s autoencoder, which can be used to train the same network on topologically different proteins. We demonstrate these new features with the help of three different molecular dynamics data sets in the context of the ubiquitin system.

## Graphical abstract

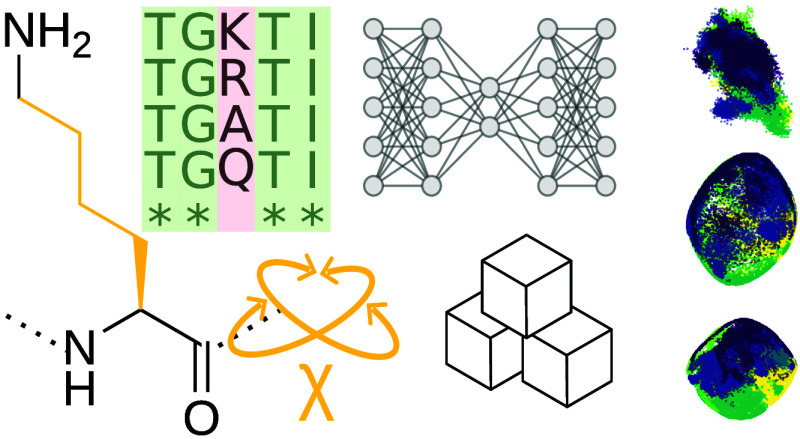

## 1 Introduction

The simulation times accessible by Molecular Dynamics (MD) have made steady advancements. This can be attributed not only to the increased power of available hardware but also to a constant optimization of the underlying algorithms. Especially, offloading increasingly more routines of the MD algorithm to GPUs has allowed MD simulations to transition from supercomputers to high-performance single-node computers.^1^ The data sets created by these ever more efficient algorithms need to be analyzed by similarly efficient algorithms.

Besides a rapid increase in the sampling speed of single MD simulations, almost all MD studies employ parallel simulations of multiple replicas.^2^ Thus, time-independent ensemble analyses need to be employed.^3,4^ The analyses carried out on ensembles can range from simple frequency plots and distributions of important features up to dimensionality reduction to approximate the free energy landscape of the protein at hand and continue with Markov state analysis.^5,6^ As the data generation is constantly driven toward longer time scales using advanced sampling techniques like multiscaling^7^ and faster hardware,^8^ modern analysis for MD is not keeping pace with these developments.^9^ Future algorithms for MD analysis need to not only be highly optimized on a per-frame basis but also allow for some form of frame-based parallelization, which can then be ported to the same powerful high performance computing (HPC) architecture that is currently used for data generation.

Most analysis pipelines either follow standard protocols (root-mean-square deviation (RMSD), root-mean-square fluctuation (RMSF), solvent accessible surface area (SASA), etc.), which are highly optimized, or are problem-specific and coded by small teams to allow integration with multiple real-life experiments.^10^ The latter of these is often carried out in a serial, simulation-frame by simulation-frame manner. Methods and algorithms originally developed for the Machine Learning (ML) field of neural network (NN) training can also be employed to tackle classical problems (such as linear regression) via parallelization and vectorization.^11^ Another point where ML tools can help is when ensemble statistics are extracted for large sets of MD simulations. Classical methods such as MDS or RMSD matrices reach their limits for quantitative ensemble statistics, as they require similarity matrices that need to fit into a computer’s memory.^12^ In an NN-based approach, training can be done in batches. This facilitates infinitely large training sets because not all available data have to simultaneously fit into system memory.

EncoderMap was created to allow researchers to leverage these emerging technologies.^13^ It offers a method to reduce dimensionality using an NN autoencoder and an MDS-like cost function augmented by the sketch-map algorithm’s sigmoid scaling function.^14^ The encoder network of EncoderMap produces a low-dimensional embedding by optimizing the network weights so that differences in the high-dimensional input are preserved as low-dimensional distances. This implementation approach shares similarities with contrastive learning. While contrastive learning offers sophisticated embedding techniques employing triplet loss functions, pretraining tasks, and semi- and self-supervised training of network weights, EncoderMap augments the embedding network (encoder) with a decoder to reconstruct high-dimensional points from the latent space.^15,16^ Traditional autoencoders often optimize for reconstruction only and might produce low-quality embeddings. EncoderMap leverages embedding techniques similar to contrastive learning, combined with sketch-map’s sigmoid loss function to offer both functionalities. The produced high-quality embeddings can be clustered to obtain states from the input data, and high-dimensional samples can be generated from anywhere on the projection using the decoder network. Contrastive learning and EncoderMap alike have been successfully employed on MD simulations of ubiquitin.^17,18^

## 2 Showcases Related to the Ubiquitin System–Biological Background

The protein ubiquitin and its interactions with the proteome present a fundamental piece of cells and higher biological life. Roughly 5% of the human genome encodes enzymes for the ubiquitin system.^19^ Ubiquitin is a 76-residue protein that can be attached to other proteins via isopeptide bonds between its C-terminus and an amino group of the substrate protein. The complexity of the ubiquitin system stems from the various modifications it can undergo as well as how ubiquitin itself modifies other proteins. In this publication, we highlight the capabilities of the new EncoderMap package, using data from three different molecular simulation studies of proteins from the ubiquitin family.

M1-linked diubiquitin is a linear two-domain protein where two Ub-units are strung together. It was previously used to showcase the capabilities of EncoderMap.^17,20^

The second example protein is the ubiquitylated linker histone H1. The linker histone H1 is a nucleoprotein that can undergo ubiquitylation.^21–23^ In subsequent studies, the ubiquitylation of H1 was found to loosen the nucleoprotein complex and to lead to an increased expression of certain genes.^24^ In a computational study, MD simulations of H1 ubiquitylated at lysines K30, K41, K47, K51, K56, and K63 were conducted. The Euclidean distances between the C_α_-atoms were used as a high-dimensional input. The sketch-map algorithm was applied to reduce these high-dimensional data to two dimensions (2D). Finally, clusters were extracted from these low-dimensional projections to represent statistically significant protein folding substates. The publication also highlighted that density-based clustering approaches can fail on sketch-map projections when some points coincide.^25^

The third example protein is a K11-modified ubiquitin. The acetylation of K11 was investigated by a joint study combining experimental, spectroscopic, and MD methods.^26^ To gauge this effect, wild type (wt) ubiquitin was compared to K11Ac, K11A, K11Q (a widely used KAc surrogate), and K11R for their HDM2 (an E3 ubiquitin ligase) affinity. If the ubiquitin E3-ligase binding would be a strictly electrical effect, the order of the variants would be expected to be K11R > wt > (K11Q, K11Ac)> K11A. However, the assays revealed an order of K11R > wt > K11Q > K11A > K11Ac, which separates KAc and its surrogate glutamine far apart. It was postulated that the structures of these two proteins are widely different, which was shown using ^1^H-^15^-HSQC NMR spectroscopy. MD simulations were carried out for these proteins. A unified projection that assesses the impact of these small sequence variations on the protein structure has not been constructed, but it could be beneficial in understanding the structure and function of these proteins.

## 3 Results

### 3.1 EncoderMap’s Modules

The new release of EncoderMap provides three modules:

- **Module one (general autoencoder module)**: A neural network autoencoder with an MDS-like cost function derived from sketch-map’s sigmoid scaling function.^14^ This general module can be provided with numerical data of any kind (also allowing data from a periodic space). The data provided to this module need not necessarily stem from MD simulations.^13^ This module has in the past years been successfully applied to a variety of protein or RNA systems, with various types of input features from dihedral angles, intermolecular and intramolecular distances, to a novel graph-centrality-based featurization (Figure S1).^27–29^

- **Module two (protein MD autoencoder module)**: A second, more specialized autoencoder network that can learn protein conformations and generate new structures from low-dimensional projections. The inputs to this module are dihedral angles derived from protein conformations (Figure S2). In EncoderMap II, only backbone conformations could be provided as input and backbone conformations could be generated. This module was successfully used on diubiquitin and a part of the Ssa1 Hsp70 yeast chaperone and the heat-shock protein B8.^17,30^ The new release of EncoderMap III adds the ability to include side chains, which allows full conformations to be provided as input and also generated. Furthermore, EncoderMap III’s module two can be used with inhomogeneous data sets containing proteins with differing topologies.
- **Module three (data handling and featurization module)**: A high-performance data handling module, which assists in the organization and featurization of large MD data sets, i.e., the efficient generation of inputs for modules one and two. This module is inspired by PyEMMA and allows EncoderMap to consume out-of-memory data of infinite size.^31^ It is also built with customization in mind and can be leveraged in user-defined analyses (Figure S3).

### 3.2 Moving EncoderMap to TensorFlow 2

The TensorFlow library is one of the most widely used libraries for machine learning via neural networks.^32^ EncoderMap I and II were written using TensorFlow version 1. However, TensorFlow 1 is now marked for deprecation, and support will likely end in the future. Furthermore, TensorFlow 1 cannot straightforwardly be installed in Python environments with Python version ≥ 3.8, possibly using outdated versions of the GNU Compiler Collection (GCC) to compile the deprecated TensorFlow version from source. The migration to TensorFlow ≥ 2.15 in EncoderMap III not only ensures compatibility with modern Python environments but also enables several new features. These include modular cost functions, support for sparse tensor input to handle heterogeneous topologies in molecular data, and out-of-memory data set processing. Hence, the integration of TensorFlow 2 is a fundamental upgrade leading to enhanced analytical capabilities and performance while we keep the functionality of EncoderMap I and II (see SI Section 1 for more info). We find that the “function over session” paradigm shift from TensorFlow 1 to 2 makes EncoderMap more versatile, because:

- TensorFlow 2 eliminates global variables and uses namespaces.
- TensorFlow 2’s eager execution eliminates sessions, allowing for easier and more streamlined development, where tensors can be compared without setting up graphs and sessions.
- TensorFlow 2 offers more features, such as probability, ragged and sparse tensors, and also graphics.

Thus, inexperienced users and professionals can benefit from TensorFlow 2 in general and EncoderMap III, written with TensorFlow 2, in particular. The functionality of EncoderMap I and II is still the core of EncoderMap, while EncoderMap III introduces ways of working with complex data sets and the ability to expand and customize it.

### 3.3 Training Using Large Data Sets

An autoencoder offers a differentiable function from the input feature space to the low-dimensional projected space and back to the high-dimensional feature space (details on the architecture can be found in SI Section 4). This property bears enormous potential for the use of the resulting projections for enhanced sampling methods.^13^

However, another benefit is often overlooked: Some dimensionality reduction algorithms like MDS face limits with the size of modern data sets. MDS must construct a memory-demanding similarity matrix *D* ^*n*×*n*^ where the distance between all input samples *n* is stored. While the nonincremental implementation of principal component analysis (PCA) is not facing such extreme limitations, it is still limited in that all values need to fit into memory as a matrix *M* ^*p*×*n*^ with *p* representing the dimensionality. PCA also needs to keep sufficient memory for the additional matrices *U*, Σ, and *V*, when performing singular-value decomposition (*M* = *U*ΣV*). This results in a memory complexity of $O\left(n^{2}\right)$ for MDS and $O\left(p^{2}\right)$ for nonincremental PCA.

Sketch-map, whose sigmoidal scaling function is implemented as a cost function in EncoderMap, provides two advantages over these classical methods when they are applied on MD data. First, small fluctuations over short time scales in the high-dimensional space are scaled so that their projections will be placed closer together. As Euclidean distances in the high-dimensional input depend on the number of dimensions, these small fluctuations would otherwise be overemphasized.^33^ Second, the computational limitations of MD and PCA are overcome by only performing MDS on a limited set of landmarks before projecting the remaining points using localized similarities, which can be done in batches.^14,34^ Similarly, NN optimizations, including contrastive learning and EncoderMap III, rely on batch-based training, where only a single batch needs to fit into memory at each training step. This possibility was not fully exploited in EncoderMap I and II, as the input data was presented as an array in memory. With current trends in the size and availability of data sets, EncoderMap III implements out-of-memory data sets using the TensorFlow and xarray libraries.^35,36^ This allows the training of MD data set sizes at biologically relevant time scales while reducing memory constraints. When applied to MD data, EncoderMap III can facilitate feature extraction and trajectory analysis, providing a modular and extensible interface.

### 3.4 Visualize Training Progress

The vastness of these data sets makes it difficult to grasp the ensemble statistics. That is why dimensionality reduction is used to better understand and present the ensemble statistics in the first place. EncoderMap III offers tracking of the training progress of the neural network via intermediate low-dimensional projections. This allows one to track how points, representing a molecular conformation, are placed and spaced in the low-dimensional projection. As training progresses, these projections are refined, revealing structural features that may correlate with important biomolecular properties.

During training, the neural network shifts those arrangements into orderly structures. These structures might correlate with important molecular features such as folding states of a biomolecule or pocket accessibility in a protein–ligand system. Some molecular features might dominate, so that they form larger structures, while others are still ordered on a local level. As an example, MD simulations of ubiquitylated linker histone (a data set composed of 1188 individual trajectories corresponding to different initial structures and different ubiquitylation sites on the histone subunit) were chosen and the same Euclidean distances as high-dimensional input as outlined by Sawade et al.^25^ were provided as high-dimensional input vectors. The scatter plots of low-dimensional projections of intermediate steps can also be colored by user-provided variables that further characterize the simulation frames (Figure 1). Three variables were chosen exemplarily to highlight this new capability and how it can assist in understanding EncoderMap projections. The first exemplary variable used is the ID of the individual trajectory a data point belongs to (as a unique identifier that discerns the trajectories started from different initial structures). During the network training, the arrangement of points shifts. Some trajectories are more spread across the projection surface. The second variable to use for coloring is the center of geometry (COG) distance between the ubiquitin and histone subunits of H1Ub. It becomes apparent that this single feature does not describe the system very well. Using the COG distance for arguments of structural distinction would be futile. Using the index of the ubiquitylated lysine as the third way of coloring the low-dimensional projections, however, shows that the network pushes every H1Ub species into its own region, showing the structural uniqueness of these proteins.

**Figure 1:**
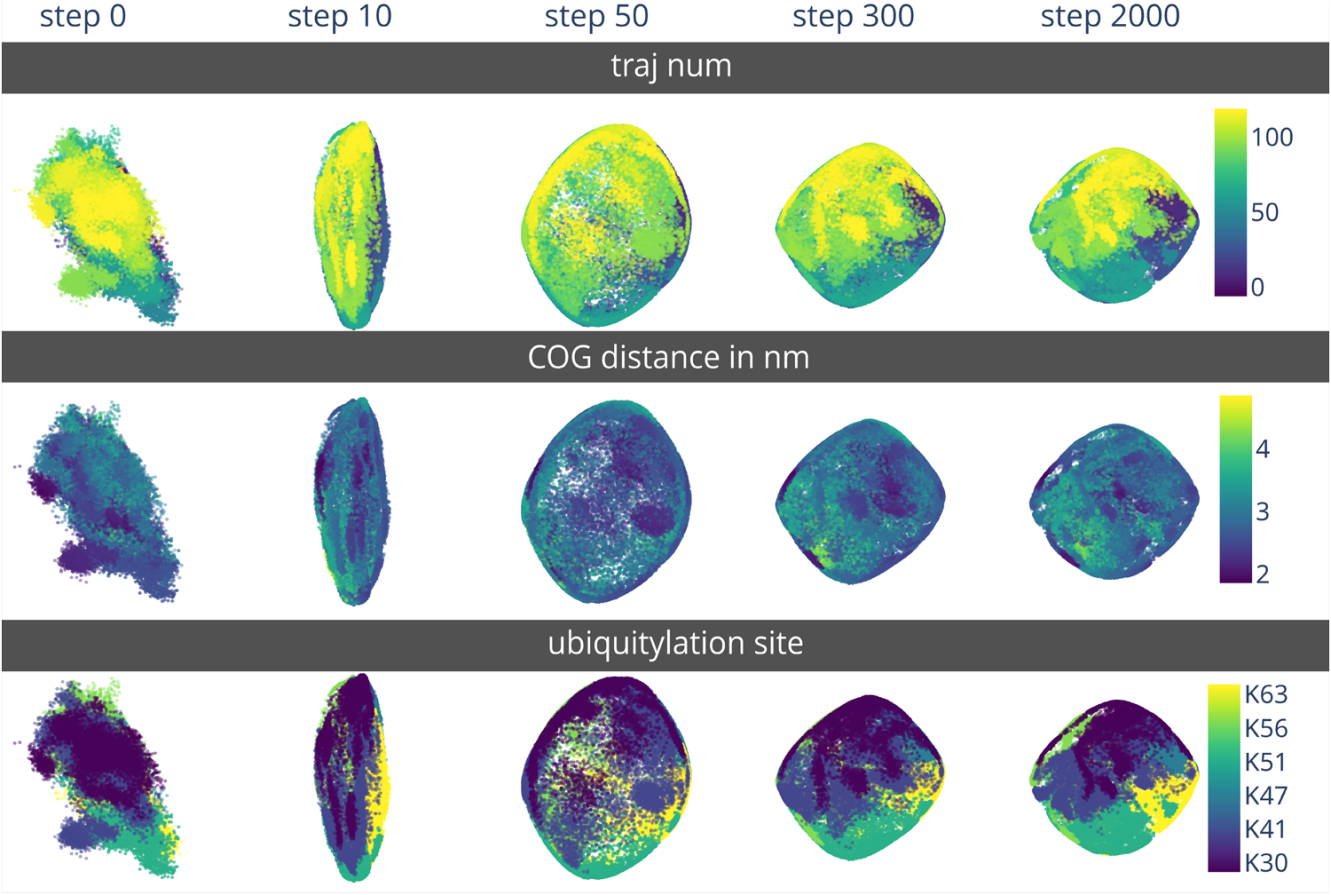
Examples of low-dimensional projections during the training of EncoderMap. The high-dimensional input data was taken from MD simulations described in a publication by Sawade et al.^25^ The low-dimensional projections can be compared with Figure 3 in the same publication. Module one was trained on this data and 2-dimensional projections of the input molecular conformations were obtained. In these projections, a point in the 2-dimensional projection represents one molecular conformation sampled by an MD simulation of ubiquitylated linker histone H1 (H1Ub). From left to right, the number of training steps increases. The projections in every column are identical in their (*x*,*y*)-coordinates. The new EncoderMap version allows viewing and logging such intermediate projections during training and allows custom colors for the points. Three exemplary colorations were chosen. Top row: Points are colored according to trajectory number. Middle row: Points are colored according to COG distance. Bottom row: Points are colored according to ubiquitylated lysine of the H1 protein.

Additionally, this new version of EncoderMap includes an RMSD metric, which can be used to observe the training progress, specifically in module two (protein MD autoencoder module), and assess convergence of the cost functions. While the minimization of loss functions is certainly the main task in neural network training, additional metrics can help when trying to gauge performance. The root-mean-square deviation of atomic coordinates is a common descriptor to compare two molecular conformations with identical topology. The Kabsch–Umeyama algorithm is implemented in EncoderMap III’s module two to calculate the RMSD distances of input Cartesian coordinates and generated coordinates.^37–39^ A distribution and mean RMSDs will be logged by EncoderMap III during training (Figure 2). Being able to log intermediate training steps’ projections and the well-known RMSD metric during the training can help immensely when assessing training progression.

**Figure 2:**
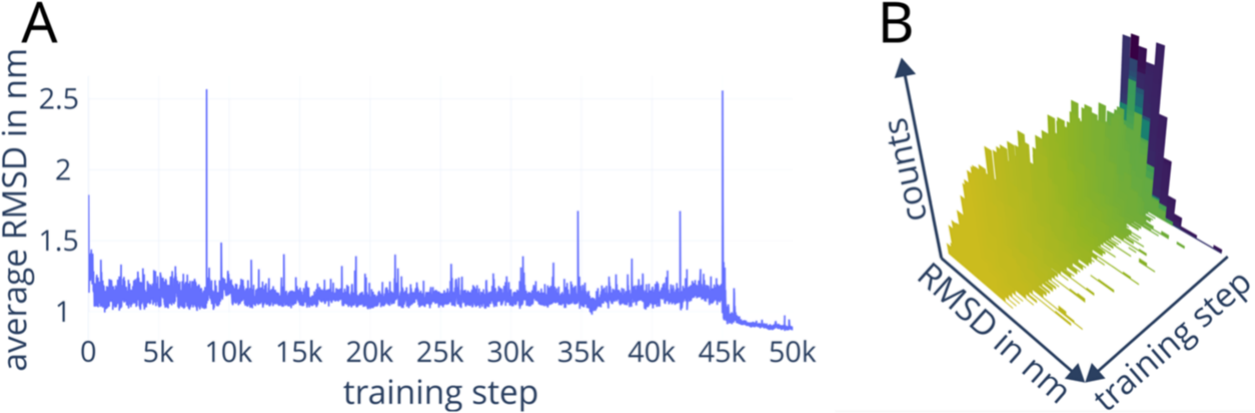
Examples of the RMSD metric that can be used to visualize the training progression of the conformational reconstruction. For these graphs, M1-connected diubiquitin was provided to module two (protein MD autoencoder module) and the neural network was trained using the Adam optimizer. The average RMSD per training step can be logged by employing this metric (A) as well as distributions of the RMSD values for each training batch (B). In panel (A), a rapid decrease in the average RMSD between input and output is depicted. It shows that the network replaces the random starting weights with sufficient weights to minimize the loss functions. The decrease at 45,000 training steps is due to the Cartesian cost (eq S37) that is only added for the last 5000 steps. The color of the frequency plots in (B) denotes training progression (dark purple: start, yellow: finish). The height of the columns represents how many samples in a batch have a corresponding RMSD distance between the input conformations and generated conformations.

### 3.5 Modularity

As stated, EncoderMap III uses version 2 of the TensorFlow library and thus can leverage the modularity of this library. EncoderMap II is a rather monolithic code that executes all ML operations in a single Python class. Thus, the user had limited room to adjust the layout of the network, computation of the cost functions, and the training. In EncoderMap III, most of these operations are represented as building blocks (via Python classes and functions) that can be combined and tuned. The sigmoid weighted distance cost function, adopted from the sketch-map algorithm, is a central component in EncoderMap. It is used in two cost functions: the so-called “distance cost” (eq S18) and the “Cartesian” distance cost (eq S30). The distance cost mimics the sketch-map approach by computing batch-pairwise distances between the high-dimensional input samples and compares it to the batch-pairwise distances of the low-dimensional encoder outputs (SI Section 3: eq S18). Both are weighted using a sigmoid function before the mean of the squared difference of these symmetric matrices is used as a scalar cost. The sigmoid cost function penalizes small distances, which in the input space can relate to small molecular fluctuations, and large distances, which might arise from the high-dimensional topology of the input space. This is done to scale the medium distance in which conformational shifts happen in molecules. If the underlying data do not stem from MD, there might be some arguments not to use this cost function or any cost function at all. In EncoderMap III, additional cost functions can be easily implemented by writing Python functions. An MDS-like cost function without these weighting factors (eq 1), or a triplet-like cost function, that compares triplets of input values and latent outputs (eq S34), can be implemented with a few lines of code (see EncoderMap’s documentation on GitHub).

1 $$C_{\mathrm{MDS}}=\frac{1}{n^{2}}\sum_{i}^{n}\sum_{j}^{n}{\left(R_{\mathrm{Highd}}^{n\times n}-r_{\mathrm{lowd}}^{n\times n}\right)}^{2}$$

Furthermore, this cost function can be completely turned off to remove even more constraints on the low-dimensional embedding. The H1Ub system is used as an example. Three variations of module one (general autoencoder module) are trained with the C_α_ distances also used in Sawade et al.^25^ The networks are trained with a combination of cost functions. Keeping all other parameters identical, the sketch-map cost function (eq S18 and Figure 3I–L) can be completely turned off (Figure 3A–D), or replaced by an MDS-like cost function (eq 1 and Figure 3E–H). After training, these three networks are used to project the complete H1Ub data set into 2-dimensional maps (Figure 3A,E,I). It becomes apparent that the sketch-map cost function results in more defined density basins than the other two cost functions. To compare these three cost functions (or the lack thereof in the “autoencoder” case), a density basin is extracted from each projection. These so-called clusters represent protein folding substates of the input MD data set. The Highd traces (Figure 3C,G,K) visualize the input data of each cluster (Figure 3D,H,L), respectively. Each column represents one input feature vector. The colors represent the actual input values (yellow low, blue high). However, the actual values are less important than the overall homogeneity of these visualizations. It directly correlates with the structural homogeneity of the selected clusters. The blue basins in the projections were obtained without any MDS-like cost function (Figure 3B). The high-dimensional traces (Figure 3C) show that the input data from these points is rather irregular when compared to the other selected clusters (orange from the MDS cost implementation and green from the sketch-map cost implementation; Figure 3F,G,J,K). Extracting 10 exemplary structures from the blue cluster, it becomes apparent that these points–while placed closely together by the NN–are structurally not as similar as their distance in the projection suggests. This can be further observed by highlighting the same blue cluster in the projection generated from an NN trained with the MDS-like cost function (eq S18 and Figure 3F). Here, the blue cluster is more spread out over the low-dimensional projection. When selecting a cluster from Figure 3F, plotting its high-dimensional input data (Figure 3G) and the secondary structure of a subset of selected points (Figure 3H), it becomes apparent that this cluster is much more structurally uniform. The distance in low-dimensional projection space better resembles the structural information on the MD data set. Furthermore, the “autoencoder” NN using no MDS-like cost function placed the points of the orange cluster over a wide range in projection space (Figure 3B, orange points). The sketch-map cost function can produce an even better embedding of structural differences with respect to distance in the low-dimensional space because it was specifically tuned for this. In summary, EncoderMap III’s modularity allows one to explore different approaches to NN autoencoders.

**Figure 3:**
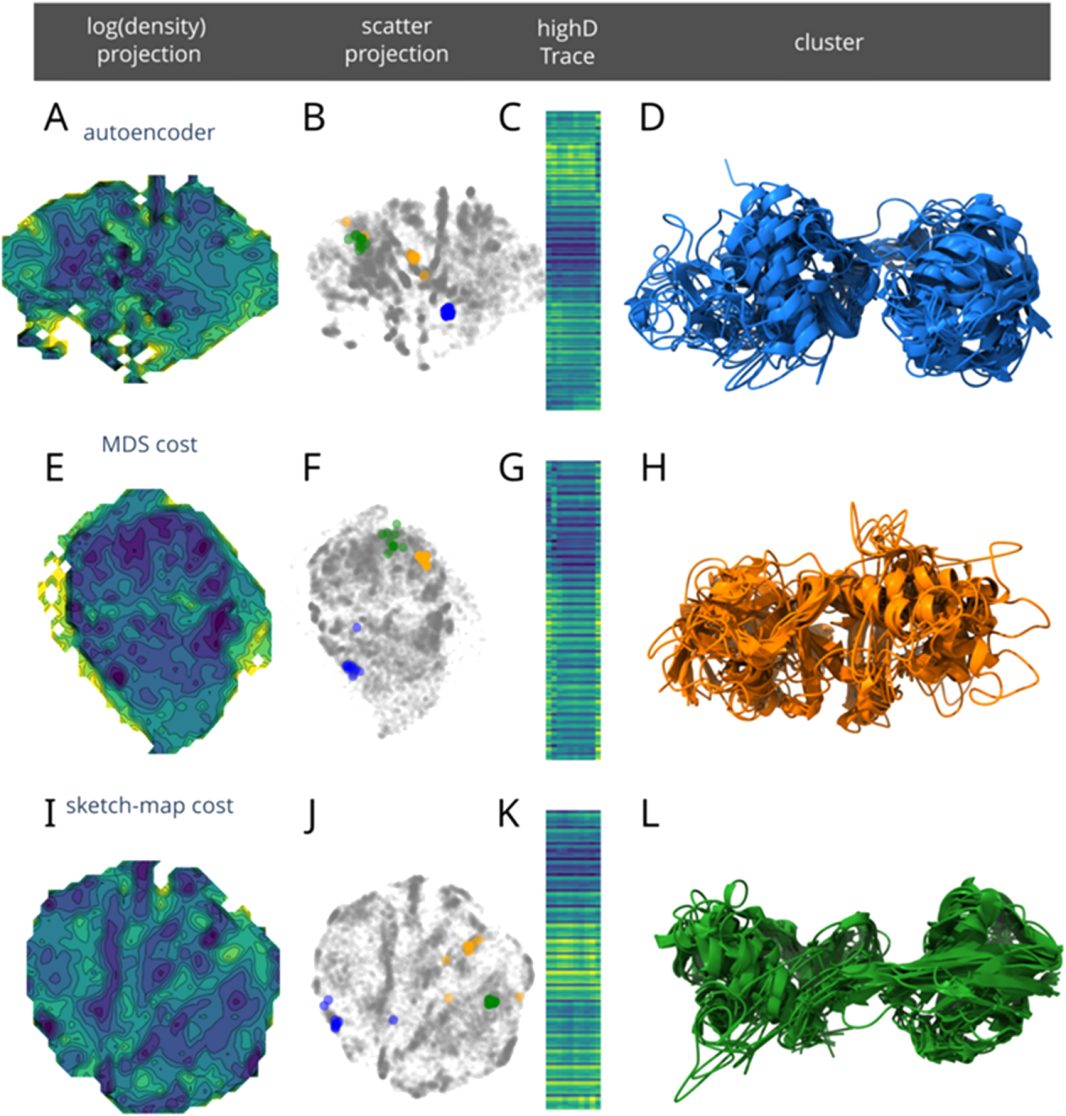
Comparison of projections of the H1Ub data obtained by using different cost functions in module one. For the top row (A–D), no cost function comparing the bottleneck output (Figure S2) with the NN input was used. For the middle row (E–H), an MDS-like cost function according to eq 1 was implemented. For the bottom row (I–L) EncoderMap’s original sigmoid-scaled cost function was used (eq S18). The high-dimensional traces (C, G, and K) can be used in addition to the secondary structure renders (D, H, and L) to gauge the heterogeneity of the conformations contained within a cluster. For details, see SI Section 3.6.

### 3.6 Generating Protein Conformations from Various Topologies

#### 3.6.1 Side Chain Dihedrals Can be Used as Input Features in Module 2

EncoderMap II introduced a way of reconstructing Cartesian coordinates from dihedral angles, angles, and bond lengths. However, this procedure was only applied to the backbone (the N, C_α_, and carboxylic C atoms). While the coordinates of the oxygen and hydrogen in an amide bond can be derived by rigid transformations from these generated Cartesian coordinates, the information on the side chains was lost. Most programs could render the reconstructed backbone from these atomic coordinates, but full molecular conformations could not be reconstructed. In EncoderMap III, a feature is added to include the side chain dihedral angles as inputs for the neural network. A complete protein can be provided as training input and a complete protein can be reconstructed from the low-dimensional encoded space. Instead of just feeding the dihedral angles of the backbone through the autoencoder network, the architecture of EncoderMap III concatenates the backbone angles, the backbone dihedrals, and the side chain dihedrals (Figure S2).

#### 3.6.2 Different (Bio)­molecule Topologies Can be Provided as Training Input

Besides allowing for side chain dihedral angles to be used as input features, another addition allows EncoderMap III to use different protein topologies as training input. This is useful because protein sequences can exhibit varying degrees of conservation inside a single organism or across species. One example of high evolutionary conservation is the protein ubiquitin, which, except for 3 different residues, is identical between humans and yeast.^40^ Other H1 proteins, like the aforementioned linker histone, are in humans expressed by 11 genes, which result in a family of five proteins, H1.0 to H1.5.^41^ While the globular domains of these 5 proteins are somewhat conserved, the N- and C-terminal tail regions are quite different in sequence and overall length.^42^ However, as protein function arises from sequence, each subtype of the histone family has been investigated for its different roles in homeostasis. It was discovered that different H1 types are enriched in different tissues.^43^ In terms of MD, different proteins with different sequences provide different topologies; a hierarchical structure of chains, residues, and atoms. If a lysine residue is replaced by a glycine residue, the side chain dihedrals χ_1_ to χ_4_ are no longer present at this position of the chain. Other examples of sequence variations include:

- Point mutations, different topologies due to an exchange of a single amino acid, which, for example, occur on the neurofibromin 1 and 2 genes.^44^
- Protein families and homologues, different topologies due to sequence variations, such as the H1 histone family with members H1.0 to H1.5.^42^
- Artificial mutations, e.g., inclusion of non-natural amino acids, which can be used to study protein functions in assays.^26^

The K11-modified ubiquitin data set offers similar but different sequences. The data set encompasses simulations of wt, K11Ac, K11A, K11Q, and K11R ubiquitin. The proteins differ in their feature space due to the different lengths of the 11th residue side chain (Figure 4).

**Figure 4:**
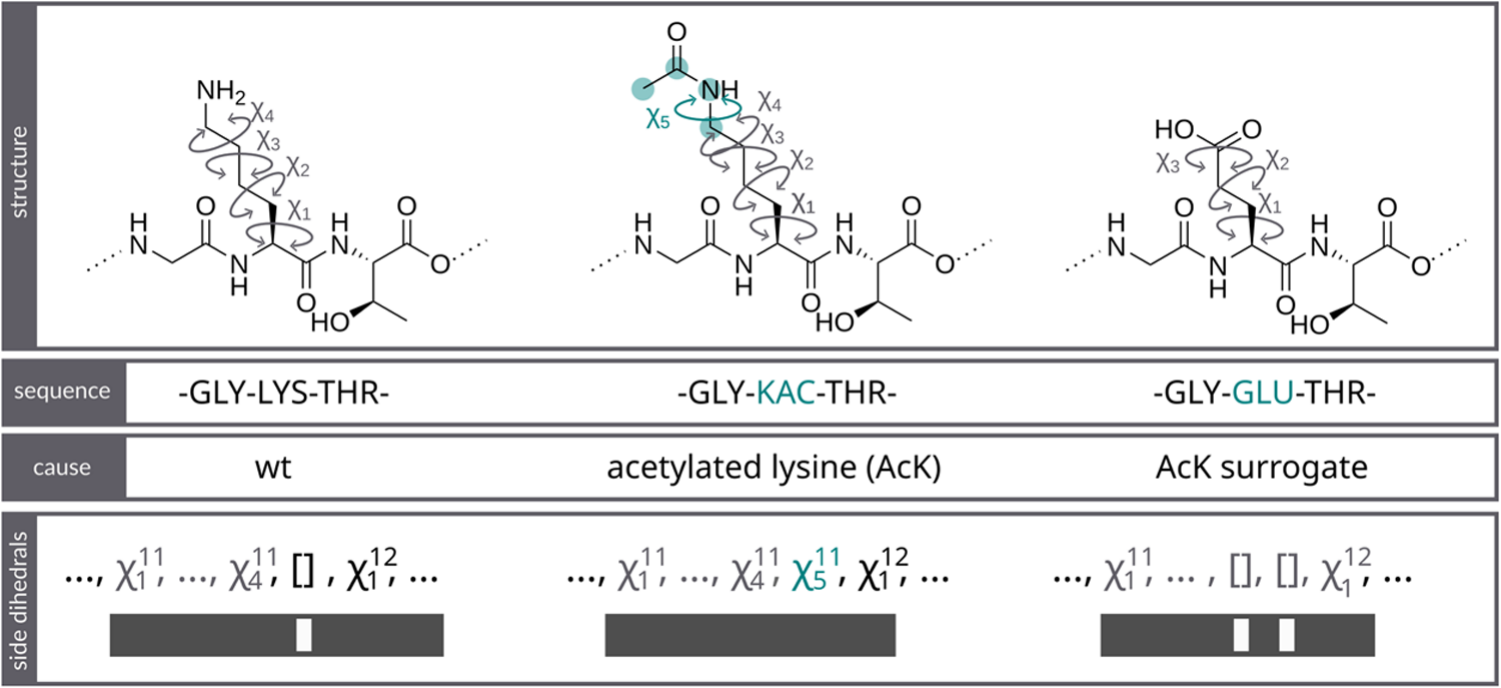
Example sequences of ubiquitin wt (left column), K11Ac (middle column), and K11Q (right column). Differences in sequences and feature space are highlighted in green. Only the K11Ac protein can provide a χ_5_ angle for the residue 11. The wt has 4 χ angles, and the K11Q mutant has 3 χ angles at this position. These topological differences lead to feature vectors of different lengths, which can not be used as input for NN training when only dense inputs are accepted. Thus, EncoderMap III allows inputs to be sparse (bottom row), where some values are undefined (represented by empty brackets).

Such data sets could previously not be trained using NNs. The new version of EncoderMap allows proteins of different lengths and side chain compositions to be used as input features. This is performed in two steps: EncoderMap’s featurization module (Figure S3) aligns the topologies and extracts the coordinates, distances, angles and dihedral angles for all input trajectories and labels them using general labels (χ_1_ of first residue, distance between N and C_α_ in third residue, etc.). This labeled data can contain NaN values for topologies that do not exhibit certain features. Both the general autoencoder module (Figure S1), as well as the protein MD autoencoder (Figure S2) employ sparse matrix multiplication as an additional input layer to allow these data to be used as training input. Using the K11-modified ubiquitin simulations, which exhibit topological differences as input to the encoder network of EncoderMap’s MD autoencoder architecture will produce a low-dimensional embedding of the input conformations (Figure 5 lowd projection). From this projection, it can be seen that the different topologies generally occupy the same regions in the low-dimensional embedded space and, thus, are also structurally similar. However, there are regions only occupied by the wt form of the protein. But generally speaking, the whole projection seems to be available to all protein forms. To make assumptions about the HDM2 binding ability of these proteins, further studies are required. However, this projection can be seen as a first step. From that projection, a cluster of points can be selected, and the corresponding structures rendered. An RMSD alignment must be carried out to overlay the chosen conformations into a bundle of structures. Some considerations need to be made when aligning the molecular structures, as they do not contain the same number of atoms. Thus, a subset of atoms needs to be selected for alignment. (Figure 5 structure selection). A Markov-type correlation of all protein isoforms and their interconnecting rate steps can be easily achieved from this projection. The decoder part can just as easily work with these inhomogeneous data as the encoder. Here, the user needs to specify which topology should be used when generating high-dimensional conformations from the projection. Based on that selection, the correct features are selected from the dense output and the conformations are reconstructed. As an example, a path is drawn in the low-dimensional projection and structures are generated from the (*x*, *y*) coordinates of this path’s points (Figure 5 path drawing and generation from path). This will generate a trajectory (represented as a movie roll in Figure 5) where the time-axis corresponds to the points along the path. The conformations are generated, including the side chain dihedrals and based on the selection of the user. Exemplarily, K11C, wt, and K11Ac structures are generated.

**Figure 5:**
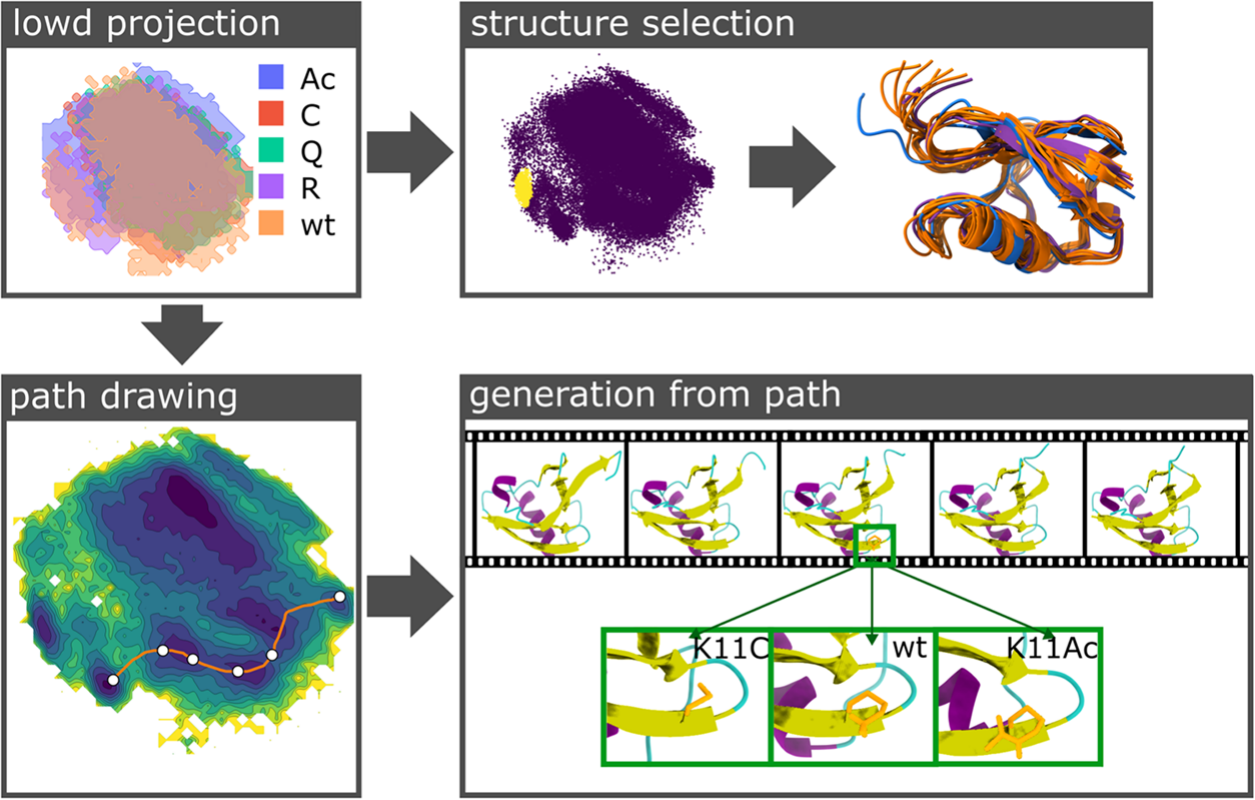
Low-dimensional projections can be interactively explored with EncoderMap. The low-dimensional projection obtained after training the neural network of module two (protein MD autoencoder module), colored according to the topology of the input trajectories (i.e., the K11 mutation), shows the first low-dimensional projection using molecular features from simulations with different topologies. From this low-dimensional projection, a cluster of points (yellow points) can be selected, and the corresponding molecular conformations can be attributed to each point. As closeness in low-dimensional space relates to structural similarity, these points originate from similar conformations. Here, the cluster consists of conformations from the wt ubiquitin (orange secondary structure render), the K11R mutant (purple secondary structure render), and the K11Ac mutant (blue secondary structure render). This structure selection does not employ the decoder part of the neural network. The decoder can be used by first defining a path in the low-dimensional projection and then using the decoder to generate new molecular conformations from the points along the path. In this case, the white points in the low-dimensional projection are rebuilt into molecular conformations (movie). It is also possible to choose the topology when decoding molecular conformations, resulting in different side chains (orange; e.g., K11C, wt, K11Ac).

To further demonstrate the versatility of EncoderMap III, we have also applied this method to topologically heterogeneous polyaspartic and polyglutamic acid simulations. These studies show that EncoderMap III can successfully cluster conformational states even for sequences with greater topological variations, a task where traditional methods like PCA fall short. These additional examples, which are provided in our GitHub repository, further showcase the broad applicability of EncoderMap III.

## 4 Conclusions

Exploring low-dimensional map projections offers an intuitive way of summarizing and understanding large, high-dimensional data sets. Whether these data sets arise from MD simulations or other sources, EncoderMap can be employed on them. The dimensionality reduction method of EncoderMap was coded into a user-friendly tool. With the additions and the update to the new TensorFlow version presented in this publication, EncoderMap will continue to be a unique method of dimensionality reduction. Our code additions allow users to work with EncoderMap in new ways, customize it if they need additional functionality, and ultimately understand what happens in the NN during the training process. The ability to visualize the training progress also allows for an assessment of whether a low-dimensional projection converges. The additions in the specialized modules two and three allow researchers to swiftly extract features from large MD data sets and present them as training data to EncoderMap. EncoderMap III presents a significant methodological advancement over its predecessors. In addition to updating the underlying software framework, EncoderMap III integrates novel features such as the incorporation of side chain dihedrals, sparse tensor handling, and modular cost function design, which collectively enhance the analysis of complex, heterogeneous biomolecular systems. These innovations address limitations that were inherent in both classical dimensionality reduction methods and previous EncoderMap versions. EncoderMap can be downloaded on GitHub: https://github.com/AG-Peter/EncoderMap.

## Supplementary Material



## Data Availability

The code of EncoderMap is openly available on GitHub at https://github.com/AG-Peter/encodermap. The analysis code for this publication and further tutorials can be found on the EncoderMap homepage https://ag-peter.github.io/encodermap/. These tutorials also contain examples where EncoderMap is used to investigate other proteins than ubiquitin (e.g., the combined training of topologically diverse polyaspartic acid and polyglutamic acid proteins). The MD data can be obtained from the University of Konstanz data repository KonDATA (also see SI Section 6).

## Notes

Additional details on backward compatibility, detailed explanations of cost functions, neural network architecture of general autoencoder and protein MD autoencoder, data handling, and featurization capabilities of EncoderMap as well as simulation details (PDF)
