# Supplementary material for: EncoderMap III: A Dimensionality Reduction Package for Feature Exploration in Molecular Simulations
Source: J Chem Inf Model. 2025 Aug 20;65(17):9000–8. doi: 10.1021/acs.jcim.5c00887 (PMC12421662; doi:10.1021/acs.jcim.5c00887)
Supplement: Supplementary file 1 [file ci5c00887_si_001.pdf]

EncoderMap III: A dimensionality reduction package for feature exploration in molecular simulations

Kevin Sawade,* Tobias Lemke, and Christine Peter*

Department of Chemistry, University of Konstanz, Universitätsstr. 10, D-78457 Konstanz, Germany

E-mail: kevin.sawade@uni-konstanz.de; christine.peter@uni-konstanz.de

Supporting Information Available

1 Old code still available

To ensure reproducibility and not invalidate Machine Learning (ML) models trained with EncoderMap II, we have included a compatibility layer in EncoderMap III. EncoderMap II code can still be used by importing `from encodermap import encodermap_tf1 as em` for as long as TensorFlow offers backwards compatibility to TensorFlow 1 inside TensorFlow 2.

2 Module one (general autoencoder module) architecture

The architecture of the neural network in module one follows the standard autoencoder architecture and was introduced in the first EncoderMap publication.[1]

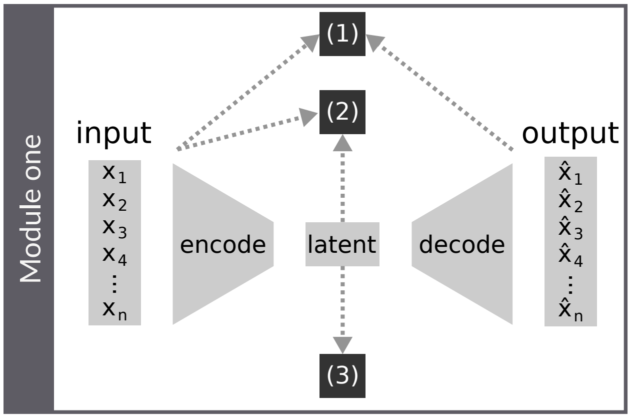

Figure S1: Neural network architecture of module one (general autoencoder module). The input can be any numeric data (periodic like angles, or non-periodic like distances). The main cost functions are the auto cost (1) (Equation (36)), the distance cost (2) (Equation (18)), and the center cost (3) (Equation (35)).

The autoencoder network consists of a series of fully-connected (also called dense) layers, a central latent layer, and a decoder part that reproduces the same dimensionality as the input layer. The latent layer is often set to two units, but more can be implemented. Except for the bottleneck and the output layer, all layers employ the hyperbolic tangent (tanh) activation function, which confines the outputs of these intermediate layers to the interval $(-1, 1)$. The training of the neural network (NN) built by EncoderMap is an integral part of the process to extract meaning from large Molecular Dynamics (MD) datasets. EncoderMap's training is a regression task where several cost functions are used to calculate gradients and apply those gradients to the layer weights and biases. The weights and biases are initialized by picking random values from distributions. The cost functions are minimized using an adaptive stochastic gradient descent algorithm (Adam).[2]

3 Cost Functions in EncoderMap III

This section outlines the cost functions in EncoderMap in general and EncoderMap III in particular.

3.1 Nomenclature

The input to the autoencoder network usually has more than 2 dimensions. In the following discussion, we will denote high-dimensional variables and functions using upper-case letters (Y, R, SIG). These can be either high-dimensional inputs to the encoder part of the neural network or output of the decoder part (Figure S2). The bottleneck neurons yield low-dimensional output. Thus, variables and functions use lower-case letters (y, r, sig) for the low-dimensional output. The number of samples in a batch n, and the number of amino acids of a protein N present exceptions to this notation. We will also be differentiating between Euclidean-pairwise and batch-pairwise distances. The Euclidean-pairwise distances $D^{N\times N}$ can be obtained from a list of points A of cardinality $|A| = N$. The list is given by:

$$A = (x_1, x_2, ..., x_N)\, \forall x \in \mathbb{R}^n \tag{1}$$

By computing the Euclidean distance $D_{i,j}$ between any two points x_i and x_j:

$$D_{i,j} = \|x_i - x_j\| \tag{2}$$

And then constructing the hollow ($D_{i,j} = 0$ for $i = j$) and symmetric ($D_{i,j} = D_{j,i}$) matrix $D^{N\times N}$ like so:

$$D^{N\times N} = \begin{bmatrix} D_{1,1} & D_{1,2} & \cdots & D_{1,N} \\ D_{2,1} & D_{2,2} & \cdots & D_{2,N} \\ \vdots & \vdots & \ddots & \\ D_{N,1} & D_{N,2} & & D_{N,N} \end{bmatrix} \tag{3}$$

In contrast, the batch-pairwise distances $R^{n\times n}$ and $r^{n\times n}$ can be calculated in either the high-dimensional input and output, or the low-dimensional latent space. Take a batch Z of n samples (these samples can be points in $\mathbb{R}^3$, but don't have to).

$$Z = Z^{(1)}, Z^{(2)}, ..., Z^{(n)} \tag{4}$$

The batch-pairwise distances $R^{n\times n}$ are calculated via:

$$R^{n\times n} = \begin{bmatrix} \|Z^{(1)} - Z^{(1)}\| & \|Z^{(1)} - Z^{(2)}\| & \cdots & \|Z^{(1)} - Z^{(n)}\| \\ \|Z^{(2)} - Z^{(1)}\| & \|Z^{(2)} - Z^{(2)}\| & \cdots & \|Z^{(2)} - Z^{(n)}\| \\ \vdots & \vdots & \ddots & \\ \|Z^{(n)} - Z^{(1)}\| & \|Z^{(n)} - Z^{(2)}\| & & \|Z^{(n)} - Z^{(n)}\| \end{bmatrix} \tag{5}$$

3.2 Distance cost

The "distance cost" in EncoderMap's module one is computed from the batch-pairwise distances of the high-dimensional input samples ($R^{n\times n}_{\text{Highd}}$, Equation (10)) and the low-dimensional output of the encoder ($r^{n\times n}_{\text{lowd}}$, Equation (14)). During a training step, the network receives a batch of n high-dimensional samples. Each sample $Y^{(i)}$ is denoted by a superscript (i) with $1 \leq (i) \leq n$, so that a high-dimensional batch Y is a list of n samples:

$$Y = (Y^{(1)}, Y^{(2)}, Y^{(3)}, ..., Y^{(n)}) \tag{6}$$

A linear protein with N amino acids provides $3N - 3$ backbone dihedral angles (ψ, ω, ϕ) each within a periodic space of $[0, 2\pi)$. Every dihedral angle is defined by 4 atoms. In the following examples, a subscript denotes the amino-acid number of an atom. So, N_1 is the backbone nitrogen atom of the first amino acid. As another example, $C_{\alpha 5}$ is the alpha carbon atom of the fifth amino acid. The dihedral angles are defined as follows:

- ψ_1: N_1, $C_{\alpha 1}$, C_1, N_2
- ω_1: $C_{\alpha 1}$, C_1, N_2, $C_{\alpha 2}$
- ϕ_2: C_1, N_2, $C_{\alpha 2}$, C_2

- ...
- ϕ_N: C_{N-1}, N_N, $C_{\alpha N}$, C_N

The subscript to the (ψ, ω, ϕ) angles also denotes to which amino acid an angle belongs. Every amino acid, except the first and last, contributes one of each dihedral angle type. The first amino acid in the chain contributes only two angles (ψ_1 and ω_1). The last amino acid of a chain only contributes one ϕ angle. Thus, the total number of backbone dihedrals of N amino acids is $3N-3$. A single high-dimensional sample $Y^{(i)}$ is a set of $3N-3$ angles:

$$Y^{(i)} = (Y^{(i)}_{\psi_1}, Y^{(i)}_{\omega_1}, ..., Y^{(i)}_{\phi_N}) \tag{7}$$

The difference $R^{(i,j)}$ between two samples $Y^{(i)}$ and $Y^{(j)}$ can then be calculated via Equation (8).

$$R^{(i,j)} = \|Y^{(i)} - Y^{(j)}\| = \sqrt{\left(Y^{(i)}_{\psi_1} - Y^{(j)}_{\psi_1}\right)^2 + \left(Y^{(i)}_{\omega_1} - Y^{(j)}_{\omega_1}\right)^2 + \cdots + \left(Y^{(i)}_{\phi_N} - Y^{(j)}_{\phi_N}\right)^2} \tag{8}$$

The periodic boundary conditions for any torsion angle m, $0 \leq Y^{(i)}_m - Y^{(j)}_m < \pi$ are enforced element-wise via Equation (9).

$$Y^{(i)}_m - Y^{(j)}_m \equiv \min\left(|Y^{(i)}_m - Y^{(j)}_m|, 2\pi - |Y^{(i)}_m - Y^{(j)}_m|\right) \tag{9}$$

The $R^{(i,j)}$ constitute the batch-pairwise distances matrix $R^{n\times n}_{\text{Highd}}$:

$$R^{n\times n}_{\text{Highd}} = \begin{bmatrix} R^{(1,1)} & R^{(1,2)} & \cdots & R^{(1,n)} \\ R^{(2,1)} & R^{(2,2)} & \cdots & R^{(2,n)} \\ \vdots & \vdots & \ddots & \\ R^{(n,1)} & R^{(n,2)} & & R^{(n,n)} \end{bmatrix} \tag{10}$$

Similar relations can be sketched for the low-dimensional encoder output y, which is a batch of n low-dimensional outputs:

$$y = (y^{(1)}, y^{(2)}, y^{(3)}, ..., y^{(n)}) \tag{11}$$

However, as the output of the encoder is usually 2-dimensional, the output only has two scalar values ($y_1^{(i)}$ and $y_2^{(i)}$), so that $y^{(i)}$ is a list of two values:

$$y^{(i)} = (y_1^{(i)}, y_2^{(i)}) \tag{12}$$

The distances of the low-dimensional encoder output $r^{(i,j)}$ of two points $y^{(i)}$ and $y^{(j)}$ for an architecture with 2 neurons in the bottleneck layer can be computed via Equation (13).

$$r^{(i,j)} = \|y^{(i)} - y^{(j)}\| = \sqrt{\left(y_1^{(i)} - y_1^{(j)}\right)^2 + \left(y_2^{(i)} - y_2^{(j)}\right)^2} \tag{13}$$

Which is the same as the Euclidean distance of two points in $\mathbb{R}^2$. No periodic boundary conditions need to be enforced for the low-dimensional data, and $r_{\text{lowd}}^{n \times n}$ can be calculated via Equation (14).

$$r_{\text{lowd}}^{n \times n} = \begin{bmatrix} r^{(1,1)} & r^{(1,2)} & \dots & r^{(1,n)} \\ r^{(2,1)} & r^{(2,2)} & \dots & r^{(2,n)} \\ \vdots & \vdots & \ddots & \\ r^{(n,1)} & r^{(n,2)} & & r^{(n,n)} \end{bmatrix} \tag{14}$$

These batch-pairwise distances are weighted by a high- and low-dimensional sigmoid function (SIG and sig), respectively. They take each three hyperparameters (Σ, A, B) for the high-dimensional SIG function and (σ, a, b) for the low-dimensional sig function. The high-dimensional SIG function is a function in $R \in \mathbb{R}_{\geq 0}$, and the low-dimensional sig function is a function in $r \in \mathbb{R}_{\geq 0}$.

$$\mathrm{SIG}_{\Sigma,A,B}(R) = 1 - \left(1 + \left(2^{A/B} - 1\right)\left(\frac{R}{\Sigma}\right)^{A}\right)^{-B/A} \tag{15}$$

$$\mathrm{sig}_{\sigma,a,b}(r) = 1 - \left(1 + \left(2^{a/b} - 1\right)\left(\frac{r}{\sigma}\right)^{a}\right)^{-b/a} \tag{16}$$

Plugging in the batch-pairwise distances ($R^{n\times n}_{\mathrm{Highd}}, r^{n\times n}_{\mathrm{lowd}}$) into Equation (16) yields $S^{n\times n}$ via Equation (17).

$$S^{n\times n} = \left(\mathrm{SIG}\left(R^{n\times n}_{\mathrm{Highd}}\right) \; - \mathrm{sig}\left(r^{n\times n}_{\mathrm{lowd}}\right)\right)^{2} \tag{17}$$

The arithmetic mean of all elements of $S^{n\times n}$ gives the "distance cost" C_{distance}.

$$C_{\mathrm{distance}} = \frac{1}{n^2}\sum_{i}^{n}\sum_{j}^{n} S^{n\times n} \tag{18}$$

3.3 Cartesian distance cost

The "Cartesian distance cost", which is only available in the more specialized module two, uses different high-dimensional batch-pairwise distances. Here, the batch-pairwise distances between the condensed C_α-atom Euclidean-pairwise distances are used as high-dimensional input. Let us again consider a linear protein with N amino acids. Each amino acid has an alpha carbon atom (C_α), so that we can define its position by C_α. A protein has multiple amino acids and thus multiple alpha carbon positions ($C_{\alpha,1}$, $C_{\alpha,2}$, $C_{\alpha,3}$, ..., $C_{\alpha,N}$). The distance $D_{k,l}$ between two C_α atoms ($C_{\alpha,k}$ and $C_{\alpha,l}$) is given by the Euclidean distance:

$$D_{k,l} = \|C_{\alpha,k} - C_{\alpha,l}\| = \sqrt{\left(C_{\alpha,k,x} - C_{\alpha,l,x}\right)^2 + \left(C_{\alpha,k,y} - C_{\alpha,l,y}\right)^2 + \left(C_{\alpha,k,z} - C_{\alpha,l,z}\right)^2} \tag{19}$$

where $C_{\alpha,k,z}$ is the z-coordinate of the kth C_α atom of a protein. The matrix of pairwise distances $D^{N\times N}$ is given by:

$$D^{N\times N} = \begin{bmatrix} D_{1,1} & D_{1,2} & \cdots & D_{1,N} \\ D_{2,1} & D_{2,2} & \cdots & D_{2,N} \\ \vdots & \vdots & \ddots & \\ D_{N,1} & D_{N,2} & & D_{N,N} \end{bmatrix} \tag{20}$$

This matrix is, similar to Equation (3), hollow and symmetric. Thus, we can create a condensed representation X by only selecting elements $D_{k,l}$ for $k < l$:

$$X = \left(D_{1,2}, D_{1,3}, ..., D_{1,N}, D_{2,3}, D_{2,4}, ..., D_{2,N}, ..., D_{N-1,N}\right) = \left(D_{k,l}\right), \text{for } k < l \tag{21}$$

This is a vector containing $\binom{N}{2}$ elements. These vectors represent internal coordinates (rotationally and translationally invariant) of a single molecular conformation. The network is still provided with a batch of n samples. Thus, we have to correct Equation (21) to highlight the fact that it is computed for a single simulation frame. We again use the superscript (i) to denote the condensed pairwise distances of a single high-dimensional sample $X^{(i)}$.

$$X^{(i)} = \left(D_{k,l}^{(i)}\right), \text{for } k < l \tag{22}$$

The batch-pairwise distances $R_{\text{cart}}^{(i,j)}$ (where "cart" stands for Cartesian coordinates) of two high-dimensional samples $X^{(i)}$ and $X^{(j)}$ can be computed from Equation (25),

$$R_{\text{cart}}^{(i,j)} = \|X^{(i)} - X^{(j)}\| \tag{23}$$

$$= \sqrt{\left(X_1^{(i)} - X_1^{(j)}\right)^2 + \left(X_2^{(i)} - X_2^{(j)}\right)^2 + \cdots + \left(X_{\binom{N}{2}}^{(i)} - X_{\binom{N}{2}}^{(j)}\right)^2} \tag{24}$$

$$= \sqrt{\left(D_{1,2}^{(i)} - D_{1,2}^{(j)}\right)^2 + \left(D_{1,3}^{(i)} - D_{1,3}^{(j)}\right)^2 + \cdots + \left(D_{2,N}^{(i)} - D_{2,N}^{(j)}\right)^2 + \cdots + \left(X_{N,-1,N}^{(i)} - X_{N-1,N}^{(j)}\right)^2} \tag{25}$$

A batch with n elements yields $R_{\text{Highd,cart}}^{n\times n}$ via Equation (26).

$$R_{\text{Highd,cart}}^{n\times n} = \begin{bmatrix} R_{\text{cart}}^{(1,1)} & R_{\text{cart}}^{(1,2)} & \cdots & R_{\text{cart}}^{(1,n)} \\ R_{\text{cart}}^{(2,1)} & R_{\text{cart}}^{(2,2)} & \cdots & R_{\text{cart}}^{(2,n)} \\ \vdots & \vdots & \ddots & \\ R_{\text{cart}}^{(n,1)} & R_{\text{cart}}^{(n,2)} & & R_{\text{cart}}^{(n,n)} \end{bmatrix} \tag{26}$$

When computing the "Cartesian distance cost", the same $r_{\text{lowd}}^{n\times n}$ (Equation (14)) can be used. However, $R_{\text{Highd,cart}}^{n\times n}$ and $r_{\text{lowd}}^{n\times n}$ are weighted by sigmoid functions with different parameters (SIG_{cart} and sig_{cart}) compared to the "distance cost". These functions require 6 hyperparameters (different from the ones described in Equation (16)): $(\Sigma_{\text{cart}}, A_{\text{cart}}, B_{\text{cart}})$ for the high-dimensional SIG_{cart} function and $(\sigma_{\text{cart}}, a_{\text{cart}}, b_{\text{cart}})$ for the low-dimensional sig_{cart}.

$$\text{SIG}_{\Sigma_{\text{cart}},A_{\text{cart}},B_{\text{cart}}}(R) = 1 - \left(1 + \left(2^{A_{\text{cart}}/B_{\text{cart}}} - 1\right)\left(\frac{R}{\Sigma_{\text{cart}}}\right)^{A_{\text{cart}}}\right)^{-B_{\text{cart}}/A_{\text{cart}}} \tag{27}$$

$$\text{sig}_{\sigma_{\text{cart}},a_{\text{cart}},b_{\text{cart}}}(r) = 1 - \left(1 + \left(2^{a_{\text{cart}}/b_{\text{cart}}} - 1\right)\left(\frac{r}{\sigma_{\text{cart}}}\right)^{a_{\text{cart}}}\right)^{-b_{\text{cart}}/a_{\text{cart}}} \tag{28}$$

Applying them yields $S_{\text{cart}}^{n\times n}$ via Equation (29).

$$S_{\text{cart}}^{n\times n} = \left(\text{SIG}_{\text{cart}}\left(R_{\text{Highd,cart}}^{n\times n}\right) - \text{sig}_{\text{cart}}\left(r_{\text{lowd}}^{n\times n}\right)\right)^2 \tag{29}$$

Finally, the "Cartesian distance cost" $C_{\text{Cartesian distance}}$ is calculated via the mean of the sum of the elements of $S_{\text{cart}}^{n \times n}$:

$$C_{\text{Cartesian distance}} = \frac{1}{n^2} \sum_{i}^{n} \sum_{j}^{n} S_{\text{cart}}^{n \times n} \tag{30}$$

3.4 MDS-like cost function

Without the sigmoid functions, a multidimensional scaling (MDS)-like cost function C_{MDS} can be implemented (Section 3.5) for module one and module two via the mean of the sum of the squared difference elements of $R_{\text{Highd}}^{n \times n}$ and $r_{\text{lowd}}^{n \times n}$ via Equation (31).

$$C_{\text{MDS}} = \frac{1}{n^2} \sum_{i}^{n} \sum_{i}^{n} \left(R_{\text{Highd}}^{n \times n} - r_{\text{lowd}}^{n \times n} \right)^2 \tag{31}$$

3.5 Triplet-like cost function

A triplet-like cost function can be implemented similarly. Here, a third point k is considered. The points i, j, and k are also called anchor, positive, and negative, respectively. The advantage can be seen in not treating pairs in isolation. For embedding of image data (let's assume hand-written digits), the triplet would be chosen so that the positive is of the same category as the anchor (both are hand-written threes) and the negative is of a different category (a hand-written four). In EncoderMap, the provided data is not labeled; we can nonetheless exploit the idea of using two pairs of points instead of one. Take again a batch of n high-dimensional inputs $Y = (Y^{(1)}, Y^{(2)}, ...Y^{(2)})$ and the corresponding encoder output $y = (y^{(1)}, y^{(2)}, ..., y^{(n)})$. We introduce a high-dimensional triplet difference $T^{(i,j,k)}$ for any tuple of three points $(Y^{(i)}, Y^{(j)}, Y^{(k)})$ selected from Y.

$$T^{(i,j,k)} = \max\left(\|Y^{(i)} - Y^{(j)}\| - \|Y^{(i)} - Y^{(k)}\| + m, 0 \right) \tag{32}$$

In Equation (32) the hyperparameter m is called the "margin". The same is done for the low-dimensional output

$$t^{(i,j,k)} = \max\left(\|y^{(i)} - y^{(j)}\| - \|y^{(i)} - y^{(k)}\| + m, 0\right) \tag{33}$$

Similarly to the construction of the $R_{\text{Highd}}^{n\times n} \in \mathbb{R}^2$ matrix (Equation (10)), we construct the $T_{\text{Highd}}^{n\times n\times n} \in \mathbb{R}^3$ and $t_{\text{lowd}}^{n\times n\times n} \in \mathbb{R}^3$ matrices which contain all possible three point parings of the batch and obtain the triplet-like cost via

$$C_{\text{triplet-like}} = \frac{1}{n^3}\sum_{i}^{n}\sum_{j}^{n}\sum_{k}^{n}\left(T_{\text{Highd}}^{n\times n\times n} - t_{\text{lowd}}^{n\times n\times n}\right)^2 \tag{34}$$

3.6 Comparison of module one cost functions on H1Ub projections

The Highd trace is obtained by plotting the 304-dimensional input data (here solvent accessible surface area (SASA) distances) on the y-axis vs the conformation. One vertical slice contains the data of one conformation. A homogeneous horizontal stripe means that this high-dimensional value is shared between all structures in the cluster. Sudden shifts in color along the horizontal axis mean that the selected structures are unique among themselves, and the selected cluster was heterogeneous. The projections A, E, and I are colored according to the negative log density of the projections, with blue representing high-density regions with similar molecular conformations and yellow representing low-density regions. The projections in B, F, and J use the same x and y coordinates, but the 2D points are colored according to selected clusters. Gray, opaque points are not considered to be in a cluster. A cluster of 2D points represents similar conformations, and thus, the quality of a projection can be gauged by the structural similarity of these 2D clusters. The blue cluster was selected from the autoencoder projection. While its points are closely together (B),

the molecular conformations exhibit a certain degree of dissimilarity (D), which can also be concluded from the high-dimensional trace (C) where some regions are different from the otherwise homogeneous horizontal bars. The orange cluster was selected from the network employing an MDS-like cost function. While the points are grouped closely in their parent projection (F), the orange cluster's 2D points are spread out in the other projections (B and J). The molecular conformations (H) and the high-dimensional trace (G) also show that the MDS-like cost function might not be best suited for projecting the SASA distances of H1Ub. Last but not least, when EncoderMap's cost function is chosen over the MDS-like cost function, the resulting cluster exhibits a great degree of coherence in projection (J), high-dimensional trace (K), and render (L).

3.7 Other cost functions

For completeness, we will also define other cost functions. The center cost function C_{center} can be obtained from $r_{lowd}^{n \times n}$ (Equation (14)) via:

$$C_{\text{center}} = \frac{1}{n^2} \sum_{i}^{n} \sum_{j}^{n} \left(r_{lowd}^{n \times n} \right)^2 \tag{35}$$

The auto cost function C_{auto} can be obtained from $R_{\text{Highd}}^{n \times n}$ (Equation (10)) and $R_{\text{Output}}^{n \times n}$, which is calculated exactly like $R_{\text{Highd}}^{n \times n}$, but using the decoder output, rather than the encoder input:

$$C_{\text{auto}} = \frac{1}{n^2} \sum_{i}^{n} \sum_{j}^{n} \left(R_{\text{Highd}}^{n \times n} - R_{\text{Output}}^{n \times n} \right)^2 \tag{36}$$

Last, but not least, the Cartesian cost function $C_{\text{Cartesian}}$ can be calculated from $R_{\text{Highd,cart}}^{n \times n}$ (Equation (26)) which is calculated from the input Cartesian coordinates and $R_{\text{Highd,cart,output}}^{n \times n}$ which is calculated exactly like $R_{\text{Highd,cart}}^{n \times n}$, but using the generated Cartesian coordinates, rather than the input:

$$C_{\text{Cartesian}} = \frac{1}{n^2} \sum_{i}^{n} \sum_{j}^{n} \left(R^{n \times n}_{\text{Highd,cart}} - R^{n \times n}_{\text{Highd,cart,output}} \right)^2 \tag{37}$$

4 Module two (protein MD autoencoder module) architecture

EncoderMap II introduced module two by using the same basic autoencoder network architecture (Figure S1) and extending it (Figure S2 upper part). EncoderMap III extends module two further by adding the sidechain torsions and sparse inputs (Figure S2 lower part).

EncoderMap's module two adds additional layers compared to module one (Figure S1). In EncoderMap II, module two generates angles, dihedrals, distances, and C_α distances from the Cartesian coordinates of the backbone atoms. The dihedrals are passed through the encoder network and produce low-dimensional coordinates. The center cost function (3) is calculated from the latent layer's output via the mean distance of each sample $y^{(i)}$ (Equation (12)) to the coordinate's origin (Equation (35)). The sigmoid-weighted sketch-map-like cost function (2) compares the pairwise distances of the high-dimensional input and the low-dimensional output of the latent layer (Equation (18)). The decoder produces generated dihedrals and can be compared with the encoder input to obtain the autoencoder cost (1) (Equation (36)). Using average angles and average distances, the generated backbone Cartesian coordinates can be obtained. EncoderMap III passes all angular data into the autoencoder network. The auto cost (Equation (36)) can be separately calculated for central dihedrals (1.1), sidechain dihedrals (1.2), and backbone angles (1.3). Due to the concatenation, the high-dimensional input for the distance cost (2) (Equation (18)) is different. Furthermore, in EncoderMap III, the generated backbone Cartesian coordinates are created from generated angles, generated central dihedrals, and average input distances. The Cartesian cost (5) (Equation (37)) and Cartesian distance cost (4) (Equation (30)) remain unchanged.

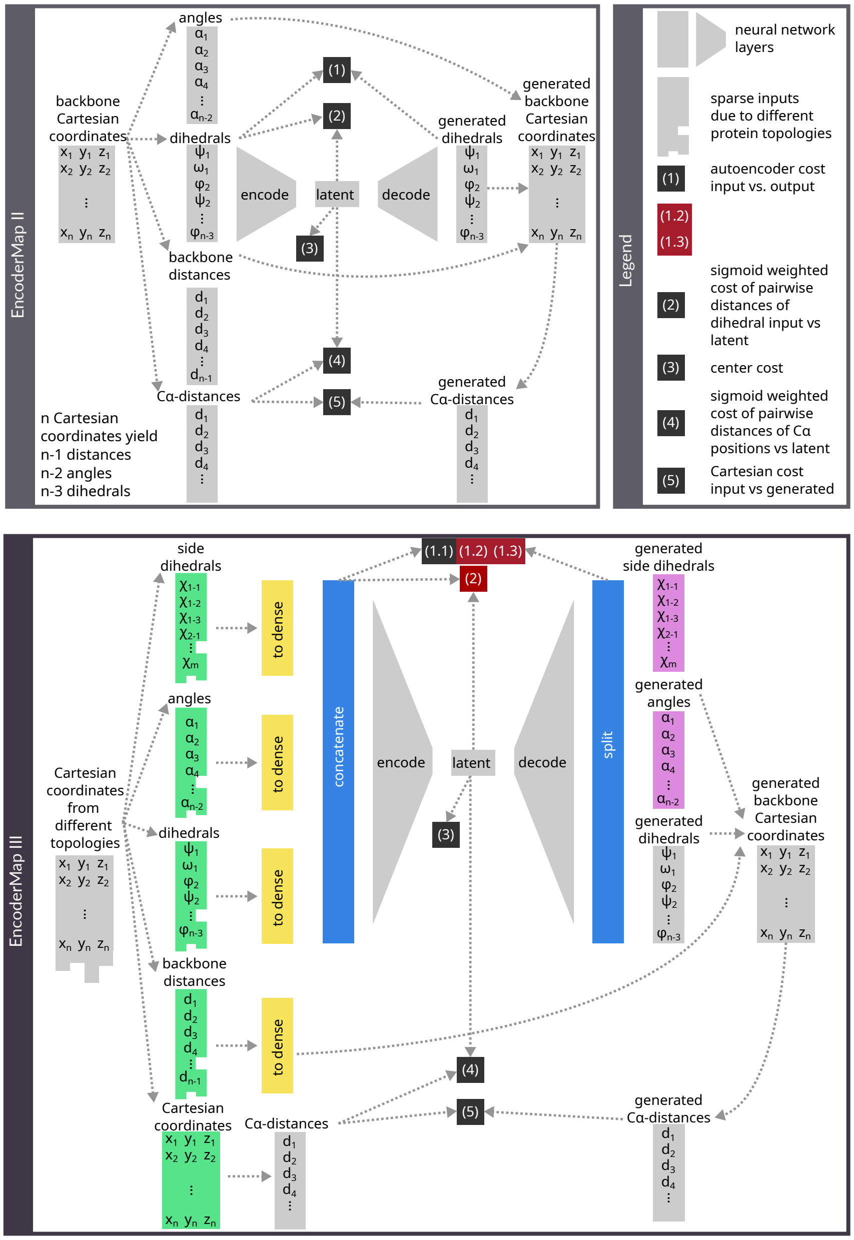

Figure S2: Comparison of neural network architectures of module two (protein MD autoencoder module) for EncoderMap II and EncoderMap III. In this overview, input and output layers are represented using rectangles. Hidden layers are represented using trapezoids, and cost functions are represented as squares. Ragged shapes represent sparse input tensors, where some values are not defined (Figure 4).

5 Module three (data and featurization module)

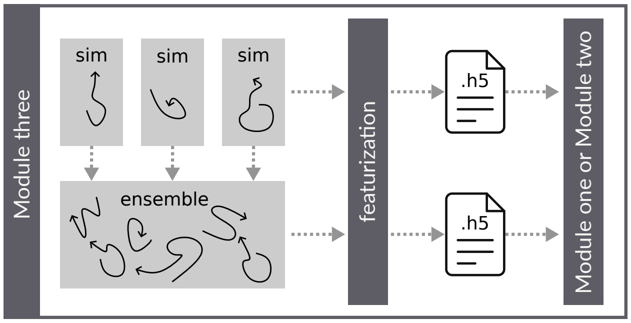

Figure S3: Overview of the capabilities of module three (data and featurization module). Simulations are represented via Python classes and contain topological (atoms, bonds) and structural (positions over time, unit cell) information. An ensemble is comprised of multiple simulations, which can all have unique topologies. A featurization module which was inspired by PyEMMA can extract features and save them into hierarchical data files using the HDF5 library.[3,4] These files can be used as a datasource for the other two modules.

6 Simulation details

The simulation details of the MD simulations used in this publication, with the corresponding publications, are given in Table S1. Further details on these datasets can be obtained from their respective publications.

Table S1: Simulation parameters. The 1GHC-Kxx in the starting structures for H1Ub refers to any of these lysine residues: K30, K41, K47, K51, K56, K63.

No.	Protein	Publication	total simulation time	program/version	forcefield	alterations
**1**	H1Ub	Sawade et al.[5]	35 µs	GROMACS/4.5.6-2022.3	GROMOS96 54A7	added iospeptide bond
**2**	Ub-K11 mutants	Kienle et al.[6]	3 µs	GROMACS/2018&2020	GROMOS96 54A7	no
**3**	M1-linked di-ubiquitin	Berg et al.[7]	600 ns	GROMACS/5	GROMOS96 54a7	no

No.	starting structures
**1**	pdb 1GHC and pdb 1UBQ, linked via isopeptide between 1GHC-Kxx and 1UBQ-M1 rotated around isopeptide χ_3
**2**	pdb 1UBQ, altered with UCSF Chimera and Vienna-PTM server
**3**	pdb 1UBQ linked using UCSF Chimera and altered position

No.	box-type	water model	equilibration	MD integrator	timestep
**1**	dodecahedral	SPC	steepest descent EM / position restrained 0.2 ps	leapfrog	2 fs
**2**	dodecahedral	SPC/E	steepest descent EM / position restrained 100 ps	leapfrog	2 fs
**3**	dodecahedral	SPC/E	energy minimization and equilibration for 3× 200 ps	leapfrog	2 fs

No.	temperature	thermostat	temp. correlation τ_T	pressure	barostat	pres. correlation τ_p
**1**	300 K	V-rescale	1.0 ps	1 atm	Berendsen	1.0 ps
**2**	300 K	V-rescale	0.1 ps	1 bar	Parinello-Rahman	2.0 ps
**3**	300 K	V-rescale		1 bar	Parinello-Rahman	

No.	bond constraints	electrostatics	long cutoff	short cutoff	neighbor step	conducted by
**1**	LINCS	PME	1.4 nm	1.0 nm	10	K. Sawade
**2**	LINCS	PME	1.4 nm		10	C. Globisch
**3**	LINCS	PME	1.4 nm			A. Berg

No.	link
**1**	`https://dx.doi.org/10.48606/99`
**2**	`https://dx.doi.org/10.48606/rx7md621k9zawfz7`
**3**	`https://dx.doi.org/10.48606/rtda48r76y1e71cc`

References

(1) Lemke, T.; Peter, C. EncoderMap: Dimensionality reduction and generation of molecule conformations. Journal of chemical theory and computation **2019**, 15, 1209–1215.

(2) Kingma, D. P.; Ba, J. Adam: A method for stochastic optimization. arXiv preprint arXiv:1412.6980 **2014**,

(3) Scherer, M. K.; Trendelkamp-Schroer, B.; Paul, F.; Pérez-Hernández, G.; Hoffmann, M.; Plattner, N.; Wehmeyer, C.; Prinz, J.-H.; Noé, F. PyEMMA 2: A Software Package for Estimation, Validation, and Analysis of Markov Models. Journal of Chemical Theory and Computation **2015**, 11, 5525–5542.

(4) Koranne, S. Hierarchical data format 5: HDF5. Handbook of open source tools **2011**, 191–200.

(5) Sawade, K.; Marx, A.; Peter, C.; Kukharenko, O. Combining molecular dynamics simulations and scoring method to computationally model ubiquitylated linker histones in chromatosomes. PLoS Computational Biology **2023**, 19, e1010531.

(6) Kienle, S. M.; Schneider, T.; Stuber, K.; Globisch, C.; Jansen, J.; Stengel, F.; Peter, C.; Marx, A.; Kovermann, M.; Scheffner, M. Electrostatic and steric effects underlie acetylation-induced changes in ubiquitin structure and function. Nature Communications **2022**, 13, 5435.

(7) Berg, A.; Kukharenko, O.; Scheffner, M.; Peter, C. Towards a molecular basis of ubiquitin signaling: A dual-scale simulation study of ubiquitin dimers. PLOS Computational Biology **2018**, 14, 1–14.
